# Supplementary material for: Tribological Behavior and Mechanism of Silane-Bridged h-BN/MoS2 Hybrid Filling Epoxy Solid Lubricant Coatings
Source: Nanomaterials (Basel). 2025 Mar 6;15(5):401. doi: 10.3390/nano15050401 (PMC11901787; doi:10.3390/nano15050401)
Supplement: Supplementary file 1 [file nanomaterials-15-00401-s001.zip › nanomaterials-3479799-supplementary.pdf]

Supporting Information

Tribological Behavior and Mechanism of Silane-Bridged h-BN/MoS_2 Hybrid Filling Epoxy Solid Lubricant Coatings

Xiaoxiao Peng [1,†], Haiyan Jing [1,†], Lan Yu [2,†], Zongdeng Wu [1], Can Su [1], Ziyu Ji [1], Junjie Shu [1], Hua Tang [2], Mingzhu Xia [1], Xifeng Xia [1,*], Wu Lei [1,*] and Qingli Hao [1,*]

[1] School of Chemistry and Chemical Engineering, Nanjing University of Science and Technology, Nanjing 210094, China; xxp0812@njust.edu.cn (X.P.); jhy24@njust.edu.cn (H.J.); zongdengwu@njust.edu.cn (Z.W.); sucan925@njust.edu.cn (C.S.); jiziyuyx@njust.edu.cn (Z.J.); shu_junjie@njust.edu.cn (J.S.); xiamzh196808@njust.edu.cn (M.X.)

[2] AECC Guizhou Honglin Aero-Engine Control Technology Corporation Ltd., Guiyang 550009, China; 19985116218@163.com (L.Y.); 18914852207@163.com (H.T.)

* Correspondence: xiaxifeng@njust.edu.cn (X.X.); leiwuhao@njust.edu.cn (W.L.); qinglihao@njust.edu.cn (Q.H.)

† These authors contributed equally to this work.

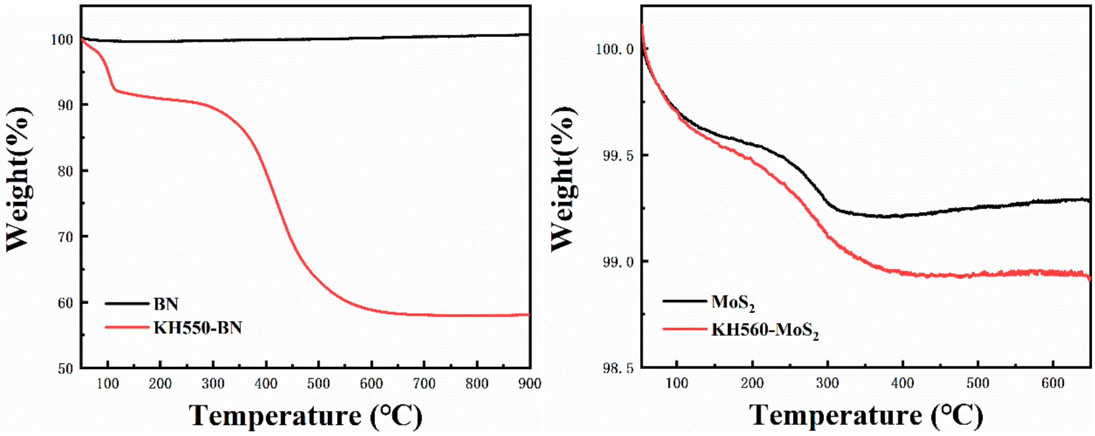

Figure S1. TGA curves of (a) h-BN and KH550-BN, (b) MoS_2 and KH560-MoS_2.

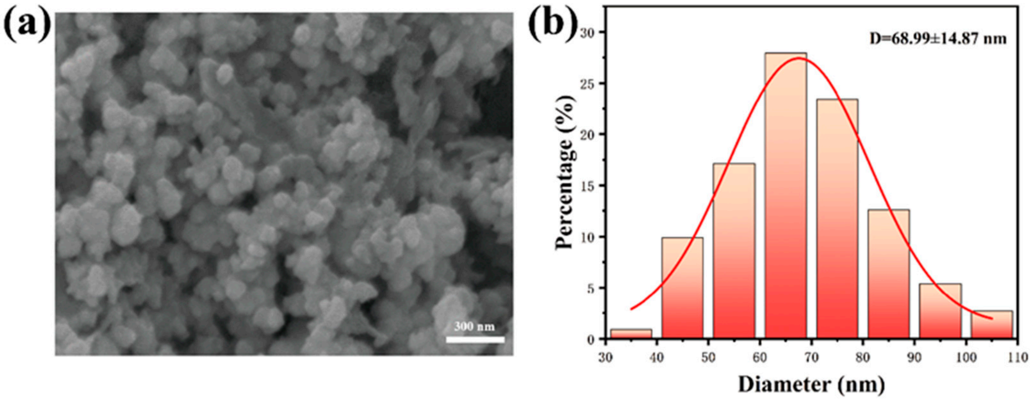

Figure S2. (a) The SEM images of h-BN/MoS_2 hybrids. (b) Particle size statistics.

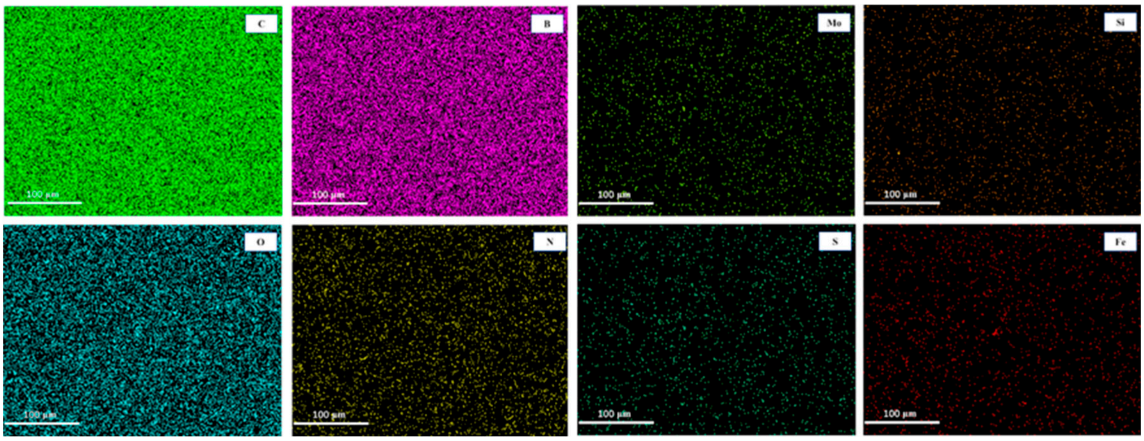

Figure S3. EDS of worn surfaces of 5 wt% h-BN/MoS_2 coating.
